# Empathetic Factors and Influences on Physical Performance: A Topical Review

**DOI:** 10.3389/fpsyg.2021.686262

**Published:** 2021-07-14

**Authors:** David G. Behm, Tori B. Carter

**Affiliations:** School of Human Kinetics and Recreation, Memorial University of Newfoundland, St. John's Newfoundland and Labrador, St. John's, NL, Canada

**Keywords:** fatigue, sport psychology, mirror neurons, athletes, training

## Abstract

Performance is dependent upon both physical and psychological factors. As a social animal, human behaviors are influenced by interactions with others. Empathy is based on social interactions and is defined as the understanding, awareness of, sensitivity to, and ability to vicariously experience the feelings, thoughts, and experience of another. There are few investigations on the influence of empathy in relation to individual and team performance and activity. There is some initial research suggesting that observing sad photos or videos or fatiguing exercise can adversely affect subsequent performance. Possible mechanisms may be attributed to mirror neurons or the affordance competition hypothesis. The relative degree of empathetic influences can be modulated by sex, age, personal familiarity, cultures and other factors. With the limited research in sport and exercise science, there is a need for more research to investigate the role of empathy on individual and team performances. The objective of this topical review was to examine the possible effects of empathy on physical performance, the potential underlying mechanisms and influencing variables moderating the association between empathy and performance?

## Introduction

Sports and exercise psychologists and physiologists may examine activity, exercise, and performance from different viewpoints, mechanisms, and measures. Nonetheless, improvements in performance are an integration of psychological and physiological influences. Psychophysiological theory would suggest that an individual's state of mind, which can be affected by their emotions, motivations, and social interactions among many other factors (Bernhardt and Signer, 2012) can influence physiological outcomes such as strength, power, and endurance. Emotions such as anger, and fear can elicit the fight or flight response activating the sympathetic nervous system to prepare for heightened levels of activity (Dhabhar, 2019). Social interactions can elicit empathetic feelings that may affect physiological responses (e.g., when a friend is sad and crying, you may start to cry, a friend is happy and laughing you may laugh in response). According to Bernhardt and Signer (2012), empathy is defined as the understanding, awareness of, sensitivity to, and ability to vicariously experience the feelings, thoughts, and experience of another. The Merriam-Webster Dictionary (2021) provides a similar definition, but adds “without having the feelings, thoughts, and experience fully communicated in an objectively explicit manner.” Another description defines empathy “as the ability to sense other people's emotions, coupled with the ability to imagine what someone else might be thinking or feeling (The Greater Good Science Center at the University of California Berkeley, 2021).” Two types of empathy have been identified. Cognitive empathy involves the ability to accurately engage in perspective taking, whereas emotional empathy is the ability to feel compassion toward others (Beadle and de la Bvega, 2009). Hence, with cognitive empathy a person may be able to identify different emotional states in another individual but without emotional empathy, they would not have an emotional reaction to the other's expressed feelings. Hence, just from observation, we can sense and respond to another individuals' pain, fatigue, distress or emotional state without them directly verbalizing their feelings to us. The notion that empathy may influence an athlete's performance has only been recently discussed in literature.

Observing others during physical activity is almost inevitable and is present in settings from recreational physical activity to professional sports and could affect physical performance. For example, neuromuscular fatigue refers to an impairment in physical performance to produce or sustain force or power (Bigland-Ritchie and Woods, 1984; Fitts and Metzger, 1993; Degens and Veerkamp, 1994; Gandevia, 2001), or a decrease in working capacity (Asmussen and Mazin, 1978), which can be associated with an increase in the actual or perceived difficulty of a task or exercise (Davis and Bailey, 1997). An increase in the perceived effort to produce or sustain a force (Enoka and Stuart, 1992), can be a symptom of decrements in physical and cognitive function (Noakes, 2012), due an increase in fatigue perception (Enoka and Stuart, 1992; Enoka and Duchateau, 2016). Our perceptions are influenced by both internal (e.g., personal past experiences) and external (e.g., social interactions) factors (Dornbusch et al., 1965; Molden et al., 2006).

The action of observing others fatiguing or in pain due to exercise has been shown in recent studies to elicit a perceptual empathic response (Paccalin and Jeannerod, 2000; Noakes, 2012; Xu et al., 2019; Yüksel et al., 2019; Astokorki et al., 2021). It is hypothesized that if a person is to observe another while exercising or view empathy-inducing images, then this natural empathy response will lead to heightened fatigue during exercise and subsequent decreases in performance. There are several proposed reasons as to why this phenomenon may occur, however the most popular rationales surround the theory of mirror neurons (Allman et al., 2011; Bernhardt and Signer, 2012) and affordance competition hypothesis (Smits et al., 2014). These theories discuss the neural processing and development of empathy and how that may apply to empathy and fatigue during exercise. Additionally, there is a dearth of research surrounding various types of exercise intervention and empathy-related responses, whether they produce performance deficits or enhancements. Thus, it is unknown if empathy has a greater effect on exercise-induced performance during anaerobic or aerobic activities. Likewise, it is unknown whether this is a long-term or short-term effect that can be overcome. For example, can the negative effects of experiencing empathy be reconciled within minutes or are the effects prolonged? Likewise, does this perceptual effect persist long enough to affect an individual's entire competition/game performance? The extent to which this response affects trained vs. untrained individuals is also unknown. Furthermore, the effect of empathy on exercise may be of interest to those wishing to further investigate team sport dynamics. In addition to individual performance responses (positive or negative), empathy may play a positive role in group or team interactions.

Emotional awareness plays a fundamental role in our emotional and social lives (Bernhardt and Signer, 2012). This emotional tendency supports adaptive social behaviors and aids in cooperation and helping qualities (Heyes, 2018). In sport, empathy promotes social behavior due to anticipated guilt and also helps to increase pro-social behaviors by promoting positive interactions with teammates and coaches (Kavussanu and Stanger, 2017). A more empathetic athlete may contribute to greater team cohesion and spirit, which could be a deciding factor for a coach when choosing between two similarly talented athletes. Further investigations regarding the plasticity of empathetic responses could be conducted to strengthen team relationships or determine ways to alleviate any empathy-related performance deficits, especially in professional sport or work environments.

Furthermore, factors such as exercise type, sex, age and individual personality differences may also play a role in understanding the relationship between empathy and performance. There is a dearth of information and these factors have yet to be fully explored in relation to performance and empathy. Even so, research surrounding these variables may provide insight into how it may affect the performance-empathy relationship. Results of this research area may allow us to understand how working in a physically active environment with others affects an individual (i.e., training, construction work, firefighters, police officers, military, athletes) and allow us to work to improve these environments to alleviate performance issues. Thus, the objective of this topical review was to examine the possible effects of empathy on athletic and work performance, potential underlying mechanisms and possible moderating variables (e.g., sex, age, culture, and others) influencing empathy.

## Mechanisms Underlying the Influencing Effects of Empathy During Exercise

Empathy allows us to share the feelings of others (Bernhardt and Signer, 2012). This process is neural-based and there are several cortical regions that are responsible for eliciting empathy, including the anterior insula (AI), sensorimotor and frontal viceromotor regions (Bernhardt and Signer, 2012; Levy et al., 2019). However, accumulating evidence from social neuroscience indicates that human empathy relies on two types of processing; lower order (automatic and sensory mechanisms) and higher-order (affect and cognition). This is modulated by a top-down control model (Levy et al., 2019). This suggests that empathetic responses are not fixed, but are modulated by a person's individual characteristics (Bernhardt and Signer, 2012). This may be further affected by hormonal differences between individuals and between the sexes (Cheng et al., 2009). The term “sex” is specifically used in this reference to reflect hormonal differences as sex refers to biological or physiological differences whereas gender is a sociological term (Canadian Institute of Health Research website)^1^. There are two major hypotheses as to how this may translate to affect exercise or work performance: the mirror neuron hypothesis and the affordance competition hypothesis.

### Mirror Neuron Hypothesis

The mirror neuron hypothesis is widely used to describe the general development of empathy in humans (Rizzolatti, 2005; Bernhardt and Signer, 2012). Mimicry or imitation behaviors have also been attributed to the mirror neuron system. A mirror neuron is a neuron that fires when acting and when observing an action performed by another; thus “mirroring” the behavior of the other person (Marcora et al., 2009). This response was observed in a study where a monkey's motor neuron activity was recorded while observing an experimenter reaching for food and compared to the monkey reaching for food. Results showed that the same motor neurons fired each time (Gallese et al., 1996). Mirror neuron activity is similar when observing others and when internally generated during motor and emotional behavior. This system permits the observer to comprehend others' behaviors or movements, with minimal complex cognitive refinement (Rizzolatti and Fabbri Destro, 2008), hence accelerating or improving the efficiency of perceiving the intention and movement of others. An efficient system for understanding movement intention and emotions would be an important advantage when attempting to determine when another person's movements are threatening, neutral, or friendly.

Mirror neurons also show activity during the understanding of other's emotions, which explains how humans succeed at understanding others actions, intentions, beliefs and feelings (Bernhardt and Signer, 2012). Mirror neuron system activation not only interprets the observed motor acts (what is happening?) but also the meaning of the entire motor actions (why is it happening?) (Rizzolatti and Fabbri Destro, 2007). They are most effectively activated when the intention of a movement is being interpreted (Rizzolatti and Fabbri Destro, 2007). This understanding of movement intent primarily involves the inferior frontal, ventral/dorsal premotor and inferior parietal regions (Rizzolatti and Fabbri Destro, 2008). The insula and anterior cingulate cortex are also involved especially when viewing another person's movement and expression of an emotion (Rizzolatti and Fabbri Destro, 2008). Disgusting odors and facial expressions are primarily perceived with the insula and anterior cingulate cortex (Rizzolatti, 2005). This “mirroring” mechanisms transform the visual information pertaining to emotion (typically communicated through facial expressions) received into our own feeling of a similar emotion (Bernhardt and Signer, 2012). Computations in the mirror neuron network in the inferior frontal, ventral/dorsal premotor and inferior parietal regions are thought to generate simulations of movements and goal-oriented actions, known as perception loops, which may serve as the basis for deriving the meaning or intention from a presented situation and for predicting future affective consequences of a stimulus (Rizzolatti and Fabbri Destro, 2007). Furthermore, neuroanatomical differences between the sexes mirror-neuron systems may affect the sensitivity to which empathy is perceived (Cheng et al., 2009). It may be hypothesized that this could translate into empathy-induced fatigue or performance decrements when observing others exercise to fatigue or sustain an injury. Viewing someone becoming fatigued or in pain may trigger an empathetic response within an individual leading to performance impairments. On the other hand, superlative performances by teammates (e.g., overcoming fatigue or an individual to score or prevent a goal) may help induce performance enhancements by the observing individual.

Involved with the mirror neuron system, Allman et al. (2011) discuss von Economo neurons (spindle cells) located in the fronto-insular cortex and anterior limbic area, which are involved with the awareness of self and others and decision making under uncertain conditions. The fronto-insular cortex and anterior limbic areas respond to facial expressions especially with decision tasks involving a high degree of uncertainty (Ullsperger and von Cramon, 2004). Neurons in the anterior insula and cingulate cortex are activated by feelings of empathy (Singer et al., 2004a) and involved with the control of goal-directed behaviors (Dosenbach et al., 2007) in response to social error (e.g., suffering of others) and negative feedback (e.g., negative facial expressions) (Gehring et al., 1993, Dehaene et al., 1994). These neurons are not only activated by negative environments or social errors. The anterior insular and anterior cingulate cortex can also respond to positive social signals such as love and trust (Bartels and Zeki, 2004; Singer et al., 2004b). Thus, these areas identify and respond to both negative and positive social environments or situations, which could either hamper performance or engender prosocial (i.e., team) dynamics.

### Affordance Competition Hypothesis

The affordance competition hypothesis is based on the direct experience of meaningful environmental events and the neuropsychological process that ensues due to this interpretation (Gibson, 1977; Cisek and Kalaska, 2010; Smits et al., 2014). The hypothesis holds that human behavior in a natural environment involves continuous and simultaneous processes of interactions between environmental stimuli and an individual's capabilities and needs. According to Cisek (2007), the brain processes sensory information to identify the possible actions or physical responses. With further sensory information, the decision becomes biased to one response. Hence, the choice of behavior requires a continual competition between the available opportunities and action demands. Both mechanisms underlying empathy; the mirror neuron hypothesis and affordance competition hypothesis, are able to coexist and allow for a greater explanation of empathy related influences in exercise and performance.

Affordance competition has been used to explain the concept of pacing and decision making in sport (Smits et al., 2014). However, it may also be able to explain empathic effects on exercise. This process begins in the occipital cortex when the action is viewed (dorsal visual system), the signal is then relayed to the premotor (fronto-parietal) cortex through the dorsal visual stream (Cisek, 2007; Smits et al., 2014). Concurrently, a variety of biasing influences are provided by the prefrontal regions and the basal ganglia; which can influence the execution of the action (Cisek, 2007; Smits et al., 2014). For example, watching an opponent fatigue in a marathon race may bias the observing competitor to slow down to conserve energy or bias (motivate) them to increase their running pace to take advantage of the other person's fatigue.

It has been shown that partaking in high-intensity exercise may influence these prefrontal regions and conscious awareness, that will affect exercise performance (Edwards and Polman, 2013; Smits et al., 2014). Understanding this, it may be hypothesized that by viewing another person exercising or viewing a series of negative images may influence the premotor cortex through signals transmitted from the occipital lobe via the dorsal stream. This would cause empathy to act as a biasing agent and thus affect action execution. This process could explain either performance deficits and increased fatigue during exercise after observing others exerting effort or improved performance from the observation of exceptional efforts or performance. After observing the performance of others, the individual may initiate or adjust a pacing strategy in anticipation of the fatigue or stress seen in competitors or teammates (Smits et al., 2014). Would this prior observation improve conscious cognitive planning of their pacing strategy or make the athlete adopt an overly cautious tempo or intensity?

## Exercise-Related Variables and Empathy

### Activity Type

Current studies have investigated both aerobic and anaerobic exercise interventions in empathy-related studies. A recent study investigated endurance cycling tasks and the influence of viewing painful images on cycling performance (Astokorki et al., 2021). Recreational cyclists attended five sessions throughout which maximum aerobic capacity and time trial tasks were conducted. The subsequent three visits consisted of the participants performing time trial and fixed power tests after viewing pleasant, painful or neutral images. Results revealed that exercise-induced pain was rated higher during the fixed power tasks following viewing the painful image but not following viewing the pleasant or neutral images (Astokorki et al., 2021). Furthermore, performance was decreased in time trials following viewing of the painful images (Astokorki et al., 2021). These results may be attributed to the empathy evoked by viewing the painful images (Smits et al., 2014). Only one study has investigated strength exercise using handgrip protocols to see if watching a sad movie could affect handgrip strength performance (Tice et al., 2007). Results showed that participants who watched a sad movie produced less force than those who watched a happy movie (Tice et al., 2007). The results of these studies, both aerobic and strength (anaerobic), may be explained by the mirror neuron hypothesis (Gallese et al., 1996), and affordance competition hypothesis. Computations within the mirror neuron network generate perception loops, which serves as the basis for predicting future affective consequences (Cheng et al., 2009). Thus, viewing the negative stimuli could derive an empathetic response and causes participants to predict or anticipate negative future affective consequences while exercising. Since insular cortex and anterior limbic area neurons respond to both negative and positive social and environmental signals (Bartels and Zeki, 2004; Singer et al., 2004b), the negative images or movie could influence goal-directed behaviors (Dosenbach et al., 2007) to possibly inhibit performance. According to the affordance competition hypothesis, the experience of viewing meaningful environmental events can act as a biasing agent impacting the premotor cortex affecting performance.

Future research should directly compare anaerobic and aerobic interventions to determine if any difference exist in their susceptibility to empathy. Furthermore, does participation in different sporting events (recreation vs. competition; solo vs. team) affect the extent to which empathy is felt? How does this interaction exist for athletes who compete in multiple events (heptathletes, gymnasts)? Research should also look to uncover any differences between long-term and short-term exposure to empathy-inducing scenarios during exercise (i.e., the effects of empathy-inducing events to alter exercise performance).

The intensity of the activity may also play a role in the ability of empathy to influence exercise. Moderate-intensity activity has shown to have a direct effect on the mirror neuron system (Xu et al., 2019). It is widely known that exercise has a beneficial effect on human behavior, and recent studies have shown that empathy is no exception. It may be suggested that if exercise activates the mirror neuron system while exercising, individuals may experience increased levels of empathy while exercising. The affordance competition hypothesis suggests there would be an empathy biasing agent affecting the premotor cortex (Edwards and Polman, 2013; Smits et al., 2014). This bias accounts for the performance deficits observed during exercise following the viewing of negative images or exertion of others (Edwards and Polman, 2013; Smits et al., 2014). This information may reveal a reciprocal relationship between empathy and exercise, as they both may act upon each other in different scenarios. In other words, exercise may elevate empathetic feelings, while empathy may impact exercise performance. Researchers looking to expand upon this idea may wish to investigate differences between exercise intensities in eliciting mirror neuron activation. Studies should compare the effect upon performing varying exercise intensities after viewing empathy-inducing images or films, or even viewing teammates or peers while exercising. This may be of interest in viewing practice vs. game scenarios, or even in the comparison of play-off and championship games to the regular season. Additionally, researchers may wish to uncover if there are any differences to the extent of empathy-related deficits between recreation and competition environments. Furthermore, is there a repeated bout or exposure effect, whereby increasing exposure to empathy-inducing scenarios either inhibits or amplifies the responses? It may be best to operate these investigations as deception studies, to ensure there is no bias amongst participants. In summary, one study reported that watching sad or negative images did impair cycling time trial performances and in one study decreased handgrip strength, however the role of exercise intensity has not been clearly examined. With only two published exercise studies, further research is necessary.

## Participant-Related Variables

### Sex-Related Differences

Empathetic differences between men and women are seen throughout a variety of tasks; exercise is no exception. Women have consistently been found to be more empathetic, and show more sensitivity and emotional recognition than men (Cheng et al., 2009). These differences are attributed to neuroanatomical sex differences in the mirror neuron system that are strongly linked to empathy competence (Cheng et al., 2009; Bernhardt and Signer, 2012). The sole study on this variable was done on mice and observed their interaction with voluntary physical activity and empathy-like behaviors (Yüksel et al., 2019). It was found that both sexes had an increase in empathy-like behavior. These results were explained through increases in oxytocin levels in females, which promoted helping behaviors. However, no mechanism was identified that could explain the increase in helping behaviors for the males (Yüksel et al., 2019). This study did not attempt to understand the subsequent effects of empathy on exercise performance or fatigue but does provide us with insight as to mechanisms that may contribute. These sex differences have been attributed to differences in the neuroanatomy of the human mirror-neuron system.

Neuroimaging studies during magnetic resonance imaging (MRI) have uncovered several neural areas that relate to the processing of facial expressions and perceiving empathy, including a network of medial and lateral prefrontal, temporal, and parietal brain regions (Schulte-Rüther et al., 2008). It has been reported that women show increased levels of activity in both the right inferior frontal cortex and superior temporal sulcus while men show a higher level of activity in the left temporoparietal junction. These results detail that women recruit neural areas containing a higher density of mirror neurons than men (Schulte-Rüther et al., 2008). This explains the ability of women to better process the emotions of others and produce empathy-like behaviors and feelings (Schulte-Rüther et al., 2008). Within the context of exercise, these neuroanatomical differences could affect the level to which empathy might affect exercise performance and fatigue. Future research should examine how these differences in empathy between the sexes affect sport involvement. Is the type of chosen physical activity influenced by the individual's genetic predisposition to be more or less empathetic? Does this further explain the higher participation amongst men in more aggressive or violent sports? Furthermore, are women at an immediate disadvantage in exercise and sport due to their predisposition to be more empathetic toward others? How does this vary between solo and team sports? Research should uncover if the effects of empathy are dependent on if the individual is participating against an individual of the opposite sex. In summary, the ability of women to better process the emotions of others and produce empathy-like behaviors has been attributed to their higher density of mirror neurons (Schulte-Rüther et al., 2008), however there are no studies investigating whether this perceived difference reflects any sex-related differences in exercise performance.

### Age-Related Differences

Aging may also play a large role in empathy capacity, as older adults often have lower “cognitive” empathy (perspective taking) than younger adults due to cognitive degeneration in key brain areas (Beadle and de la Bvega, 2009). However, aging has shown to produce little effect on “emotional” empathy (ability for individuals to feel compassion toward others) (Beadle and de la Bvega, 2009). There is a dearth of research surrounding the effects of aging and empathy during exercise. It remains unknown if cognitive or emotional empathy is more prevalent in exercise-related settings. As such, the extent to which elderly persons may be affected during exercise remains unclear. Future research should isolate these variables and conduct comparative analyses in order to understand the influence of aging on empathy and exercise. Research should explore if the effect of empathy on exercise is magnified with aging, and if so, how does this affect participation of older adults in physical activity? How can an environment conducive to healthy physical activity practices be created? Is partnered activity or solo activity more beneficial if empathy is viewed as the main variable?

Early theorists suggested that young children were too egocentric and not cognitively able to experience empathy (Freud, 1958; Piaget, 1965). Current literature has suggested the opposite, showing that children are capable of showing sophisticated empathy-related behaviors. The development of empathy continues throughout childhood and into early adulthood, where it becomes stable (McDonald and Messinger, 2010). Throughout childhood, as empathy develops, there may be differences in empathetic ability that affect the relationship between empathy and exercise. To date, no studies have investigated this area of research. This research should be conducted in order to understand the role empathy may play in exercise (e.g., performance enhancement vs. fatigue, team dynamics, preference for team vs. individual sports) for young children and adolescents. The results of this could play a role in understanding participation in various competition levels of sport and dropout rates following significant events like loss or injury. In summary, there are no studies investigating if the lower cognitive empathy of older adults, or the development of empathy throughout childhood and into early adulthood plays a role in exercise performance.

### Personality-Related Differences

Empathy is a multidimensional characteristic and can be influenced by a host of external and internal factors. Several studies have confirmed that empathy is related to personality (Guilera et al., 2019). In early childhood, distinct neural circuits and perspective taking tendencies (cognitive empathy) are developed. Variations in situational characteristics, personality traits and mental disorders can modulate these circuits and tendencies, and affect the ability of an individual to empathize with others (Stietz et al., 2019). On a biological basis, individual differences in emotional empathy are negatively correlated with gray matter volume in the precuneus, inferior frontal gyrus, and anterior cingulate (Banissy et al., 2012). Of the factors attributed to personality (extraversion, emotional stability, conscientiousness, openness to experience); empathy is closely related to agreeableness (degree of personal warmth, friendliness, helpfulness, generosity, tactfulness and optimistic outlook) (Mooradian et al., 2011). Agreeableness relates closely to empathy as empathetic concern reflects the other-oriented emotion of feeling responsibility to help those in need or to be concerned for their feelings. Personality is relatively stable, but substantial and consequential personality changes can occur through all stages of life as a result of specific life experiences (Roberts and Mroczek, 2008).

These personality factors are influenced by a variety of external variables including situational and organizational factors. Although not yet studied, the components of emotional stability, openness to experience and agreeableness may increase the likelihood of being more open to external influences (e.g., athletic performance of others) that could enhance or impair performance. Certain personality traits may help promote greater social cohesion and thus positive team dynamics. The variability in participant personality may significantly affect results of studies. Research should ensure that participants complete personality screening or tests to be able to understand the impact of personality on empathy during exercise. This empathy-exercise performance relationship has yet to be identified within research and would be useful in determining individual suitability to exercise tasks or susceptibility to motivational or stressful external events.

### Cultural Differences

Another factor that plays a key role in empathy is culture. Individuals from western industrialized nations live in a more individualistic society. As such, there is a great emphasis placed upon competition and individual goals. The greater egocentric nature of some western societies contributes to valuing self-oriented behaviors over helping or empathy-based behaviors (Shaffer et al., 2020). On the contrary, children from collectivist societies and subcultures are taught to suppress individualism and work toward goals that benefit the group (Triandis, 1995; Shaffer et al., 2020). The promotion of prosocial behavior allows for empathy to be valued as a positive character trait instead of being perceived negatively (Shaffer et al., 2020). However, within each culture, there will be a broad spectrum of empathetic traits, that will also be affected by family, friends, immediate community (e.g., an individual within a small northern Inuit community vs. an individual living in a large metropolis such as New York, London, or Toronto), media and other factors. Within the context of exercise, this may be important as culture may affect the ability to express empathy toward others. Furthermore, subcultures existing within sports teams may attribute to the development or hinderance of empathy during competition. It is unknown how this may affect team and individual performances. Future research should compare the influence of empathy on exercise using participants from both collectivist and individualist societies in order to determine if culture plays a significant role in both empathy expression and empathy-induced exercise deficits. The effect of a mixture of cultures within a group or team is of emerging interest, especially in the present era where there is a greater confluence of international players on professional soccer, basketball, ice hockey and other team sports. Do these cultural empathetic differences lead to internal team conflict? Furthermore, are there distinct cultural empathetic influences on performance in individual international sport competitions such as tennis, squash, combative sports among others? For example, would an athlete from a more collectivist society be more likely to ease up on an opponent in a situation where they are overwhelming beating their opponent compared to an athlete from a more individualistic society? Unfortunately, there is no research to date comparing possible differences in individual or team performances from primarily collectivist vs. individualistic cultures.

### Familiarity Between the Empathizer and the Other

Does having connection and bonds with others make us more susceptible to the effects of empathy during exercise? The interpersonal relationship between empathizer and other has been shown to play a significant role in the extent of empathy displayed in a situation (Singer and Lamm, 2009; Bucchioni et al., 2015; Hao et al., 2019). One study uncovered that during a perspective taking activity, in-group bias was overwhelming; participants showed more empathetic responses to those in-group vs. those out-of-group (Hao et al., 2019). Furthermore, research has unveiled that connections between people play a role in empathy. Individuals are quicker to establish empathy responses for their loved ones than for strangers or for people they dislike (Bucchioni et al., 2015). This knowledge may be important in understanding team environments and the role empathy may play. Within a team, there are often strong bonds and connections formed. This may be hypothesized to increase the empathy felt toward the other teammates possibly affecting fatigue and performance. Another consideration, would be the empathetic response to a former teammate who was traded to another team and is now an opponent: could this affect the intensity of effort against the former teammate? Future research should explore the effects of familiarity on empathy during exercise. This may be beneficial to both sport and professional environments, as familiarity and strong bonds between teammates is often thought to improve performance and outcome.

## Conclusions

There is scant research on the effects of empathetic responses in relation to prior or ongoing exercise or activity. With some evidence suggesting that observing fatiguing exercise can negatively affect subsequent performance, should there be strategies put in place to limit the observance of others prior to or during competition? However, it is presently unknown whether this tactical information from observing others, would be more valuable to the athlete than the possible negative affective influence. Would prior knowledge of an individual's empathetic tendencies influence athlete selection to improve team cohesion or would a coach place a greater emphasis on a less empathetic “win at all costs” athlete? Mechanisms underlying this phenomenon may be related to mirror neurons (promote mimicry or imitation behaviors), or the affordance competition hypothesis (interactions between environmental stimuli and an individual's capabilities and needs), which may influence personality types. Empathetic differences have been generally observed between sexes (higher empathy in women), age (less cognitive empathy but lack of difference in emotional empathy with aging), cultures (more empathy with collective vs. individualistic societies) and interpersonal relationships (greater empathy for individuals within a group). These factors could influence the choice of an individual to choose team or individual sports. Empathy research can improve the comprehension of group dynamics and their effect on individuals in physically active or demanding environments. Strategies can be employed to design or alter environments to enhance performance. With the limited research in sport and exercise science, there is a strong need for further research to investigate the role of empathy on individual and team performances (Table 1).

**Table 1:** Empathetic effects on performance: research summary.

Empathy “the ability to sense other people's emotions, coupled with the ability to imagine what someone else might be thinking or feeling”	
Proposed Mechanisms	
Mirror Neuron Hypothesis	Affordance Competition Hypothesis
Moderating Variables	
Activity Type	↓ cycling time trials!!!break ↓ handgrip strength
Sex	↑ empathy with women No research on performance effects
Age	↓ cognitive empathy with elderly No research on performance effects
Personality	Relatively stable over the lifespan but still malleable with life experiences No research on performance effects
Cultural	Effect of collectivistic vs. individualistic societies No research on performance effects
Familiarity	↓ empathy toward strangers No research on performance effects
Effects on performance or team cohesion	
Enhance performance?	Impair performance?
A call to action to initiate further research in this area!	

## Author Contributions

TC conducted the literature search. Both authors were involved with writing the manuscript, read, and approved the final manuscript.

## Conflict of Interest

The authors declare that the research was conducted in the absence of any commercial or financial relationships that could be construed as a potential conflict of interest.

## Footnotes

^1^https://cihr-irsc.gc.ca/e/48642.html.

## References

[B1] Allman J. M. Tetreault N. A. Hakeem A. Y. Manaye K. F. Semendeferi K. Erwin J. M.. (2011). The von Economo neurons in fronto-insular and anterior cingulate cortex. Ann. N. Y. Acad. Sci. 1225, 59–71. 10.1111/j.1749-6632.2011.06011.x 21534993 PMC3140770

[B2] Asmussen E. Mazin B. (1978). A central nervous component in local muscular fatigue. Eur J Appl Physiol. 38, 9–15. 10.1007/BF00436748 631122

[B3] Astokorki A. Flood A. Mauger A. (2021). Images depicting human pain increase exercise-induced pain and impair endurance cycling performance. J. Sports Sci. 39, 138–146. 10.1080/02640414.2020.1809162 32809900

[B4] Banissy M. J. Kanai R. Walsh V. Rees G. (2012). Inter-individual differences in empathy are reflected in human brain structure. NeuroImage 62, 2034–2039. 10.1016/j.neuroimage.2012.05.081 22683384 PMC3778747

[B5] Bartels A. Zeki S. (2004). The neural correlates of maternal and romantic love. Neuroimage 21, 1155–1166. 10.1016/j.neuroimage.2003.11.003 15006682

[B6] Beadle J. de la Bvega C. (2009). Impact of aging on empathy: review of psychological and neural mechanisms. Front Psychol. 10:31. 10.3389/fpsyt.2019.00331 31244684 PMC6580149

[B7] Bernhardt B. Signer T. (2012). The neural basis of empathy. Annu. Rev. Neurosci. 35, 1–23. 10.1146/annurev-neuro-062111-150536 22715878

[B8] Bigland-Ritchie B. Woods J. (1984). Changes in muscle contractile properties and neural control during human muscular fatigue. Muscle Nerve 7, 691–699. 10.1002/mus.880070902 6100456

[B9] Bucchioni G. Lelard T. Ahmaidi S. Godefroy O. Krystkowiak P. Mouras H. (2015). Do we feel the same empathy for loved and hated peers? PloS ONE 10:e0125871. 10.1371/journal.pone.0125871 26024234 PMC4449017

[B10] Cheng Y. Chou K. H. Decety J. Chen I. Y. Hung D. Tzeng O. J.. (2009). Sex differences in the neuroanatomy of the human mirror-neuron system: a voxel-based morphometric investigation. Cogn. Neurosci. 158, 713–720. 10.1016/j.neuroscience.2008.10.026 19010397

[B11] Cisek P. (2007). Cortical mechanisms of action selection: the affordance competition hypothesis. Philos. Trans. R. Soc. B 362, 1585–1599. 10.1098/rstb.2007.2054 17428779 PMC2440773

[B12] Cisek P. Kalaska J. F. (2010). Neural mechanisms for interacting with a world full of action choices. Annu. Rev. Neurosci. 33, 269–298. 10.1146/annurev.neuro.051508.135409 20345247

[B13] Davis M. Bailey S. (1997). Possible mechanisms of central nervous system fatigue during exercise. Med. Sci. Sports Exerc. 29, 45–57. 10.1097/00005768-199701000-00008 9000155

[B14] Degens H. Veerkamp J. H. (1994). Changes in oxidative capacity and fatigue resistance in skeletal muscle. Inter. J. Biochem. 26, 871–878. 10.1016/0020-711X(94)90079-5 8063011

[B15] Dehaene S. Posner M. I. Tucker D. M. (1994). Localization of a neural system for error detection and compensation. Psychol. Sci. 5, 303–305. 10.1111/j.1467-9280.1994.tb00630.x

[B16] Dhabhar F. S. (2019). The power of positive stress - a complementary commentary. Stress 22, 526–529. 10.1080/10253890.2019.1634049 31339410

[B17] Dornbusch S. M. Hastorf A. H. Richardson S. A. Muzzy R. E. Vreeland R. S. (1965). The perceiver and the perceived: their relative influence on the categories of interpersonal cognition. J. Pers. Soc. Psychol. 1, 434–440 10.1037/h0021864 14328759

[B18] Dosenbach N. U. Fair D. A. Miezin F. M. Cohen A. L. Wenger K. K. Dosenbach R. A.. (2007). Distinct brain networks for adaptive and stable task control in humans. Proc. Natl. Acad. Sci. U.S.A. 104, 11073–11078. 10.1073/pnas.0704320104 17576922 PMC1904171

[B19] Edwards A. M. Polman R. (2013). Pacing and awareness: brain regulation of physical activity. Sports Med. 43, 1–8. 10.1007/s40279-013-0091-4 23990402

[B20] Enoka R. M. Duchateau J. (2016). Translating fatigue to human performance. Med. Sci. Sports Exerc. 48, 2228–2238. 10.1249/MSS.0000000000000929 27015386 PMC5035715

[B21] Enoka R. M. Stuart D. G. (1992). Neurobiology of muscle fatigue. J. Appl. Physiol. 72:1631–1648. 10.1152/jappl.1992.72.5.1631 1601767

[B22] Fitts R. H. Metzger J. M. (1993). Mechanisms of muscular fatigue. Principles Exerc Biochem. 38, 248–268. 10.1159/000422649

[B23] Freud S. (1958). Civilization and Its Discontents. New York, NY: Doubleday Anchor Books.

[B24] Gallese V. Fadiga L. Fogassi L. Rizzolatti G. (1996). Action recognition in the premotor cortex. Brain 119, 593–609. 10.1093/brain/119.2.593 8800951

[B25] Gandevia S. C. (2001). Spinal and supraspinal factors in human muscle fatigue. Physiol. Rev. 81, 1725–1789. 10.1152/physrev.2001.81.4.1725 11581501

[B26] Gehring W. J. Goss B. Coles M. G. H. Meyer D. E. Donchin E. (1993). A neural system of error detection and compensation. Psychol. Sci. 4, 385–390. 10.1111/j.1467-9280.1993.tb00586.x

[B27] Gibson J. J. (1977). The concept of affordances, in Perceiving, Acting, and Knowing, eds Shaw R. Bransford J. (Hillsdale: Erlbaum), 67–82.

[B28] Guilera T. Batalla I. Forné C. Soler-González J. (2019). Empathy and big five personality model in medical students and its relationship to gender and specialty preference: a cross-sectional study. BMC Med. Educ. 19:57. 10.1186/s12909-019-1485-2 30764878 PMC6376790

[B29] Hao Y. Nan W. Yang G. Li Q. Wu H. Liu X. (2019). Your performance is my concern: a perspective-taking competition task affects ERPs to opponent's outcomes. Front. Neurosci. 13:1162. 10.3389/fnins.2019.01162 31736696 PMC6829177

[B30] Heyes C. (2018). Empathy is not in our genes. Neurosci. Biobehav. Rev. 95, 499–507 10.1016/j.neubiorev.2018.11.001 30399356

[B31] Kavussanu M. Stanger N. (2017). Moral behavior in sport. Curr. Opin. Psychol. 16:185–192. 10.1016/j.copsyc.2017.05.010 28813348

[B32] Levy J. Yirmiya K. Goldstein A. Feldman R. (2019). The neural basis of empathy and empathetic behaviour in the context of chronic trauma. Front. Psychiatry 10:562. 10.3389/fpsyt.2019.00562 31474883 PMC6706815

[B33] Marcora S. M. Staiano W. Manning V. (2009). Mental fatigue impairs physical performance in humans. J. Appl. Physiol. 106, 857–864. 10.1152/japplphysiol.91324.2008 19131473

[B34] McDonald N. M. Messinger D. (2010). The development of empathy: how, when, and why. Free Will, Emotions, and Moral Actions: Philosophy and Neuroscience in Dialogue. pp.112–186.

[B35] Merriam-Webster Dictionary (2021). Available online at: https://www.merriam-webster.com/dictionary/empathy (accessed June 1, 2021).

[B36] Molden D. C. Plaks J. E. Dweck C. S. (2006). “Meaningful” social inferences: effects of implicit theories on inferential processes. J. Exp. Soc. Psychol. 42, 738–752. 10.1016/j.jesp.2005.11.005

[B37] Mooradian T. A. Davis M. Matzler K. (2011). Dispositional empathy and the hierarchical structure of personality. Am. J. Psychol. 124, 99–109. 10.5406/amerjpsyc.124.1.0099 21506454

[B38] Noakes T. (2012). Fatigue is a brain-derived emotion that regulates the exercise behavior to ensure the protection of whole body homeostasis. Front Physiol. 11:382. 10.3389/fphys.2012.00082 22514538 PMC3323922

[B39] Paccalin C. Jeannerod M. (2000). Changes in breathing during observation of effortful actions. Brain Res. 862, 194–200. 10.1016/S0006-8993(00)02145-4 10799685

[B40] Piaget J. (1965). The Moral Judgment of the Child. New York, NY: Harcourt Brace, 12–102.

[B41] Rizzolatti G. (2005). The mirror neuron system and its function in humans. Anat. Embryol. 210, 419–421. 10.1007/s00429-005-0039-z 16222545

[B42] Rizzolatti G. Fabbri Destro M. (2008). The mirror system and its role in social cognition. Curr. Opin. Neurobiol. 18, 179–184. 10.1016/j.conb.2008.08.001 18706501

[B43] Rizzolatti G. D. Fabbri Destro M. (2007). Focus: brain and mind. Understanding actions and the intentions of others: The basic neural mechanism. Eur. Rev. 15, 209–222. 10.1017/S1062798707000221

[B44] Roberts B. W. Mroczek D. (2008). Personality trait change in adulthood. Curr. Dir. Psychol. Sci. 17, 31–35. 10.1111/j.1467-8721.2008.00543.x 19756219 PMC2743415

[B45] Schulte-Rüther M. Markowitsch H. J. Shah N. J. Fink G. R. Piefke M. (2008). Gender differences in brain networks supporting empathy. NeuroImage 42, 393–403. 10.1016/j.neuroimage.2008.04.180 18514546

[B46] Shaffer D. Kipp K. Wood E. Willoughby T. (2020). Developmental Psychology, 5th Edn. Toronto, ON: Nelson Publishers, 43–158.

[B47] Singer T. Kiebel S. J. Winston J. S. Dolan R. J. Frith C. D. (2004a). Brain responses to the acquired moral status of faces. Neuron 41, 653–662. 10.1016/S0896-6273(04)00014-5 14980212

[B48] Singer T. Lamm C. (2009). The social neuroscience of empathy. Ann. N.Y. Acad. Sci. 1156, 81–96. 10.1111/j.1749-6632.2009.04418.x 19338504

[B49] Singer T. Seymour B. O'Doherty J. Kaube H. Dolan R. J. Frith C. D. (2004b). Empathy for pain involves the affective but not sensory components of pain. Science 303, 1157–1162. 10.1126/science.1093535 14976305

[B50] Smits B. Pepping G. J. Hettinga F. (2014). Pacing and decision making in sport and exercise: the roles of perception and action in the regulation of exercise intensity. Sports Med. 44, 763–775. 10.1007/s40279-014-0163-0 24706362

[B51] Stietz J. Jauk E. Krach S. Kanske P. (2019). Dissociating empathy from perspective-taking: evidence from intra- and inter-individual differences research. Front. Psychiatry 10:126. 10.3389/fpsyt.2019.00126 30930803 PMC6428036

[B52] The Greater Good Science Center at the University of California Berkeley (2021). https://greatergood.berkeley.edu/topic/empathy/definition

[B53] Tice D. M. Baumeister F. Schmueli D. Muraven M. (2007). Restoring the self positive affect helps improve self regulation following ego depletion. J Exp Soc Psychol. 43:379–384. 10.1016/j.jesp.2006.05.007

[B54] Triandis H. C. (1995). New Directions in Social Psychology, Individualism and Collectivism. Boulder, CO: Westview Press, 27–99.

[B55] Ullsperger M. von Cramon D. Y. (2004). Neuroimaging of performance monitoring: error detection and beyond. Cortex 40, 593–604. 10.1016/S0010-9452(08)70155-2 15505969

[B56] Xu Z. Hu M. Wang Z. Li J. Hou X. Xiang Q. (2019). The positive effect of moderate-intensity exercise on the mirror neuron system: an fNIRS study. Front. Psychol. 10:986. 10.3389/fpsyg.2019.00986 31130900 PMC6509238

[B57] Yüksel O. Ateş M. Kizildag S. Yüce Z. Koç B. Kandiş S.. (2019). Regular aerobic voluntary exercise increased oxytocin in female mice: the cause of decreased anxiety and increased empathy-like behaviors. Balkan Med. 36, 257–262. 10.4274/balkanmedj.galenos.2019.2018.12.87 31140236 PMC6711252

